# Associations of Lifestyle Behaviour and Healthy Ageing in Five Latin American and the Caribbean Countries—A 10/66 Population-Based Cohort Study

**DOI:** 10.3390/nu10111593

**Published:** 2018-10-30

**Authors:** Christina Daskalopoulou, Artemis Koukounari, José Luis Ayuso-Mateos, Martin Prince, A. Matthew Prina

**Affiliations:** 1 Department of Health Service and Population Research, Institute of Psychiatry, Psychology and Neuroscience, King’s College London, London SE5 8AF, UK; martin.prince@kcl.ac.uk (M.P.); matthew.prina@kcl.ac.uk (A.M.P.); 2 Department of Infectious Disease Epidemiology, London School of Hygiene & Tropical Medicine, Faculty of Epidemiology and Population Health, London WC1E 7HT, UK; Artemis.Koukounari@lshtm.ac.uk; 3 Department of Psychiatry, Universidad Autónoma de Madrid, 28029 Madrid, Spain; joseluis.ayuso@uam.es; 4 Instituto de Salud Carlos III, Centro de Investigación Biomedica en Red de Salud Mental (CIBERSAM), 28029 Madrid, Spain; 5 Department of Psychiatry, Hospital Universitario de La Princesa, Instituto de Investigación Sanitaria Princesa (IIS-Princesa), 28006 Madrid, Spain

**Keywords:** older adults, healthy ageing, nutrition, physical activity, lifestyle behaviour

## Abstract

Latin American and the Caribbean countries exhibit high life expectancy and projections show that they will experience the fastest growth of older people in the following years. As people live longer, it is important to maximise the opportunity to age healthily. We aimed to examine the associations of lifestyle behaviours with healthy ageing in Cuba, Dominican Republic, Peru, Mexico and Puerto Rico, part of the 10/66 study. Residents 65 years old and over (n = 10,900) were interviewed between 2003 and 2010. In the baseline survey, we measured four healthy behaviours: Physical activity, non-smoking, moderate drinking and fruits or vegetables consumption. Healthy ageing was conceptualised within the functional ability framework over a median of 4 years follow-up. Logistic models were calculated per country and then pooled together with fixed-effects meta-analysis. People engaging in physical activity and consuming fruits or vegetables had increased odds of healthy ageing in the follow-up (OR: 2.59, 95% CI: 2.20–3.03; OR: 1.24, 95% CI: 1.06–1.44, respectively). Compared with participants engaging in none or one healthy behaviour, the ORs of participants engaging in two, three or four healthy behaviours increased in a linear way (OR: 1.60, 95% CI: 1.40–1.84; OR: 2.29, 95% CI: 1.94–2.69; OR: 2.46, 95% CI: 1.54–3.92, respectively). Our findings highlight the importance of awareness of a healthy lifestyle behaviour among older people.

## 1. Introduction

The world population is ageing and by 2050 there will be more older than younger people [1]. Even though population ageing is a human achievement, there are still many health policy challenges to be addressed [2]. Increases in life expectancy have not been followed by relevant increases in disease-free years, meaning that people are living longer but are spending more years with illness and disability [3]. Living longer but in ill health could result in severe demand of public and health care resources [4]. As people experience ageing with great heterogeneity in their health pathways [5], it is crucial to identify those behaviours that are associated with a slower and healthier ageing process.

Until recently, healthy ageing research has been complicated by a lack of common definition and measurement of it [6,7]. However, the latest report on health and ageing from the World Health Organization (WHO) provided a common framework to conceptualise healthy ageing; the functional ability framework. Healthy ageing is now defined as the process of developing and maintaining the functional ability that enables well-being in older age [8]. Healthy lifestyle behaviours (for instance, never smoking, moderate alcohol consumption, physical activity and daily consumption of fruits and vegetables) have been consistently associated with better health outcomes in older people: successful ageing [9], increases in life-years spent in good health [10], a reduced risk of mortality [11,12], and of poor cognitive function [13]. Recent systematic reviews also indicated the beneficial effects of physical activity, non-smoking and of a healthy diet to healthy ageing [2,14,15].

The latter reviews also revealed the lack of evidence in low-and-middle income countries (LMICs) even though it is estimated that by 2050, 80% of the global population that is 60 years old and over will reside there [16]. Among those countries, Latin American and the Caribbean (LAC) are the ones that have been witnessing an increased life expectancy from 1990 to 2016 [17] and projections show that in the next 15 years they are to experience the fastest increase in the number of older people [1]. Dietary risk, physical inactivity, alcohol use and smoking are among the top leading factors of disability-adjusted life years (DALYs) in the area [18].

In this study, we aimed to examine the impact of four healthy lifestyle behaviours (i.e., physical activity, non-smoking, moderate alcohol consumption and consumption of fruits and vegetables), individually and in combination, on healthy ageing and survival in a cohort of people aged 65 years old and over living in five LAC countries after a follow-up period of 4 years.

## 2. Materials and Methods

### 2.1. Study Population

The 10/66 Dementia Research Group (10/66 DRG) population-based studies of ageing and dementia have been conducted in geographically defined catchment areas in LMICs (Cuba, Dominican Republic, Peru, Venezuela, Mexico, China, India and Puerto Rico) with a sample size between 1000 and 3000 (generally 2000). In this study, we used data from the baseline survey (2003–2008), including people 65 years old and over, and the follow-up assessments 3 to 5 years later (2008–2010) from Cuba (n = 3000), Dominican Republic, Peru, Mexico and Puerto Rico (n = 2000). Detailed description of the study settings is available elsewhere [19,20]. Access to the dataset can be obtained via the official website www.alz.co.uk/1066. Local ethical committees and the ethical committee of the Institute of Psychiatry of King’s College London approved the studies: Cuba-Finlay Albarran Medical Faculty of Havana Medical University Ethical Committee (January 2003, September 2006); Dominican Republic-Consejo Nacional de Bioética y Salud (CONABIOS); Mexico-Instituto Nacional de Neurología y Neurocirugía Ethics Committee (Ref: 96/07; 78/07); Peru-El Instituto de la Memoria, Depresión y Enfermedades de Riesgo (IMEDER) Ethics Committee (August 2004, May 2007); Puerto Rico-Oficina para la Protección de Participantes Humanos en Investigación (OPPHI) (Ref: FWA00005561); King’s College London-Institute of Psychiatry/SLAM NHS Trust Ethical Committee Research (ECR) (2004, Ref: 076/03), King’s College Research Ethics Committee (KREC) (February 2007, Ref: CREC/06/07/38).

### 2.2. Baseline Measures

Lifestyle behaviours were assessed based on participants’ responses to specific questionnaires. Physical activity was categorised as: “very physically active”, “fairly”, “not very”, and “not at all”. Smoking status was categorised as “current”, “former” and “never smoker”. Alcohol consumption was assessed by asking the units of alcohol drunk per week and then categorised as “no alcohol consumption”, “moderate alcohol consumption” (1–14 units per week for women; 1–21 units per week for men) and “heavy alcohol consumption” (≥15 units per week for women and ≥21 units per week for men) [21]. We assessed dietary habits by asking the number of fruit and vegetable servings in the last 3 days. In our analyses, we compared physically active participants (“very physically active” or “fairly”) with non-physically active (“not very” or “not at all”); non-smokers with ever-smokers (“current” or “former”); moderate drinkers with abstainers (no alcohol consumption) or heavy drinkers; participants with “daily consumption” of fruits and vegetables (3 or more servings the last 3 days) with “non-daily consumption” (0–2 servings the last 3 days). We also adjusted our results for socioeconomic factors: age, sex and level of education (none, some, did not complete primary, completed primary, completed secondary, tertiary).

### 2.3. Outcome Assessment

In accordance with the WHO definition of healthy ageing, we conceptualised healthy ageing within the functional ability framework [8]. We created a healthy ageing index by employing information from 26 health related question-indicators which were either self-reported or were provided by key informants. Items included information on daily disabilities and difficulties, on pain and sleep problems and on cognition abilities among others (Supplementary File S1).

To create a measurement model for healthy ageing, we employed the framework of Exploratory Factor Analysis (EFA) and Confirmatory Factor Analysis (CFA) [22]. We initially identified the number of factors needed to reproduce the latent (unobserved) construct of healthy ageing (EFA) and then we used a bifactor model to represent a single general construct of it (CFA) [23]. To establish model fit, we examined the comparative fit index (CFI) and root mean square error of approximation (RMSEA) with 90% confidence intervals (CI). We considered a model to have an acceptable fit when CFI ≥ 0.90 and RMSEA values were close or less than 0.06 [24]. We also calculated omega hierarchical (ω_H_) reliability coefficient of the bifactor structure; a high ω_H_ > 0.80 indicates that the general factor is the dominant source of systematic variance [25]. Finally, we calculated healthy ageing (factor) scores by using the regression method [26].

We transformed scores in a scale of 0–100 with higher values indicating better health. We examined 3 categories for the outcome of interest: healthy ageing, normal ageing and death at follow-up. To create these categories, we calculated the quintiles of the baseline healthy ageing score distribution and participants belonging in the three lowest fifths (i.e., 0–67.92 scores) were characterised as “normal agers”, whereas participants belonging in the two highest fifths (i.e., 67.93–100 scores) were characterised as “healthy agers”.

We performed two separate logistic regression analyses: one to estimate the odds ratios (ORs) of healthy ageing (with normal ageing and death as the non-event) and another one to estimate the ORs for survival (in which death was the non-event). We did all analyses per country and we then provided a pooled result by performing fixed-effects meta-analysis. We also computed Higgin’s I^2^ to estimate the proportion of variability across countries that accounted mostly for heterogeneity rather than sampling error [27]. Heterogeneity up to 40% is conventionally considered negligible, whereas heterogeneity more than 50% is considered moderate to substantial [28].

We also performed sensitivity analyses to assess the robustness of our findings. Firstly, we examined whether the associations of lifestyle behaviours with healthy ageing were influenced by deaths. To do this, we excluded deaths from our sample. Secondly, we performed our analyses by including the non-dichotomised lifestyle variables. Finally, we investigated whether our findings were influenced by the way we categorised participants as healthy or normal agers and if there were differences between men and women. Our analyses were performed in STATA 14.1 and Mplus 7.4 [26].

## 3. Results

The EFA indicated a latent structure of four factors and the bifactor model (χ^2^ = 4039.14, df = 273, *p*-value < 0.00l) exhibited good fit (CFI: 0.985; RMSEA: 0.040, 90%CI: 0.039–0.041). The psychometrically informative bifactor-derived statistics indicated that a strong percentage of the total score variance can be attributed to the general factor (ω_H_ = 0.85). Hence, we proceeded by considering the score of the general factor only. More information about the EFA and CFA is provided in Supplementary File S2.

In our study there were 10,900 participants in the baseline assessment. In the follow-up survey 1648 were healthy agers, 5594 normal agers, 1734 died and 1924 were either untraceable or refused to be re-interviewed. We performed analyses per country and we checked if participants included in our analyses differ from those not available in the follow-up wave (*t*-test for continuous variables, χ^2^ test for categorical). We found that participants included in our analyses in general did not differ with those excluded (Supplementary File S3). There were some statistically significant (i.e., *p*-value < 0.05) differences in Cuba and Peru regarding the dietary habits between those included in the follow-up wave and those excluded. A higher percentage of participants available in the follow-up wave endorsed daily consumption of fruits and vegetables compared to those not available (i.e., 75.4% versus 66.3% in Cuba; 82.5% versus 76.2% in Peru).

Table 1 presents the characteristics of participants characterised as healthy agers and those characterised as normal agers in the follow-up. Participants in the healthy ageing group, compared with the normal ageing group, were younger at baseline, had completed at least primary level education, were mostly physically active and endorsed daily consumption of fruits and vegetables.

The associations of each lifestyle behaviour with healthy ageing and survival at the end of the follow-up period are presented in Table 2. Compared with non-physically active participants, those who were physically active at baseline had 2.59 (95% CI: 2.20–3.03) times greater odds of being healthy agers and 2.23 (95% CI: 1.98–2.52) times greater odds of survival. Compared with people consuming two or less servings of fruits and vegetables in the last three days, people consuming three or more were more likely to experience healthy ageing (OR: 1.24, 95% CI: 1.06–1.44) and to survive at the end of the follow-up (OR: 1.13, 95% CI: 0.99–1.28). Non-smoking, compared to former or current smoking, was not associated with healthy ageing (OR: 0.95, 95% CI: 0.82–1.10) but it was positively associated with survival as never smokers had 1.17 (95% CI: 1.02–1.34) times greater odds of remaining alive in the follow-up assessment. Moderate drinking, compared to high or never drinking, was not associated with healthy ageing (OR: 1.04, 95% CI: 0.82–1.30) or survival (OR: 1.11, 95% CI: 0.90–1.37). Heterogeneity was not statistically significant in most cases (*p*-value < 0.05) indicating that the variation among countries was limited. The only significant heterogeneity was observed in the associations of physical activity with healthy ageing (I^2^: 61.3%; *p*-value: 0.035). Further investigation concluded that Puerto Rico was the country exhibiting the highest point estimate (i.e., OR: 6.30; 95% CI: 3.43–11.56). We also reproduced the pooled estimates by excluding this country and the new pooled estimates did not change the direction or strength of associations between physical activity and healthy ageing (i.e., OR: 2.42; 95% CI: 2.05–2.86; I^2^: 0.0%, *p*-value: 0.693).

**Figure 1:**
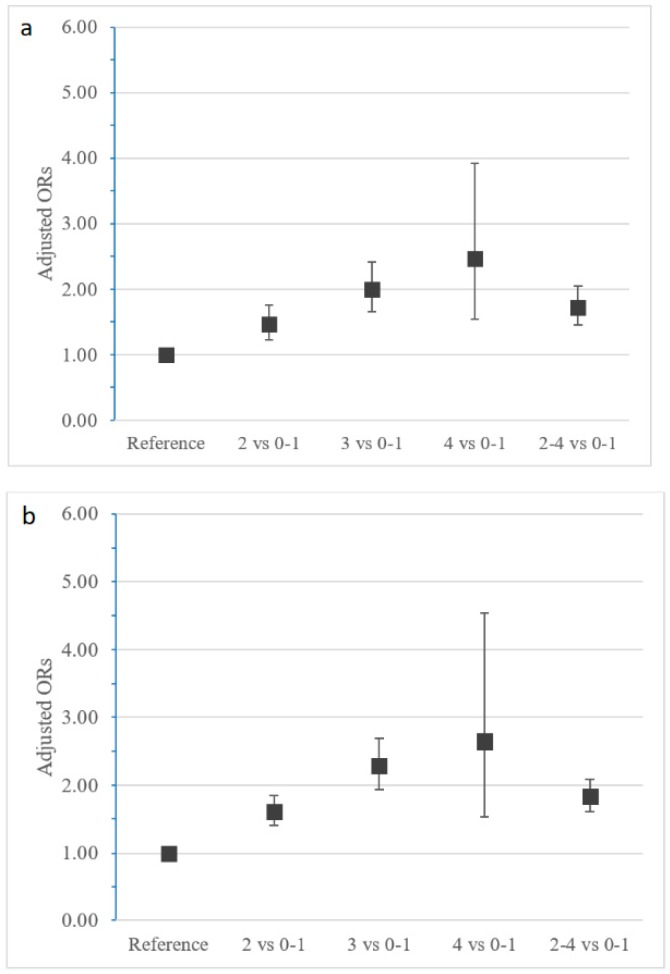
Associations of (**a**) healthy ageing and (**b**) survival with the number of healthy behaviours. Notes: ORs: Odds Ratios (pooled effects of the fixed-effects meta-analysis); models are adjusted for sex, age and education level and for all behaviours at baseline. Associations are given per individual number of healthy behaviours (two, three, four) and for two-four compared to no or one healthy behaviour (reference category). Error bars indicate 95% confidence intervals.

Participants who engaged in two-four healthy lifestyle behaviours at baseline, compared with those who engaged in none or one, exhibited increased odds of healthy ageing (OR: 1.72, 95% CI: 1.45–2.05) (Figure 1a). Additionally, the association between number of healthy behaviours and odds of healthy ageing was positive linear. More specifically, compared with participants engaging in none or one healthy behaviour, those engaging in two had 1.46 times (95% CI: 1.22–1.76) the odds of healthy ageing, those engaging in three had 2.00 times (95% CI: 1.65–2.42) the odds and those in four had 2.46 times (95% CI: 1.54–3.92) the odds. A similar beneficial association was also observed in surviving (Figure 1b). Participants engaging in two or more healthy behaviours had 1.83 times (95% CI: 1.61–2.08) the odds of surviving compared with participants that engaged in none or one healthy behaviour. A positive linear relationship was also observed; two healthy behaviours OR: 1.60, 95% CI: 1.40–1.84; three healthy behaviours OR: 2.29, 95% CI: 1.94–2.69; four healthy behaviours OR: 2.64, 95% CI: 1.53–4.54 (Table 3). Heterogeneity was not statistically significant (*p*-value < 0.05) indicating that the variability among countries was limited.

Further sensitivity analysis showed that when we excluded deaths from our sample, the associations of lifestyle behaviour with healthy ageing did not substantially change (i.e., fairly or very physically active OR: 2.28, 95% CI: 1.93–2.69; never smoking OR: 0.95, 95% CI: 0.81–1.10; moderate drinking OR: 1.05, 95% CI:0.83–1.32; daily consumption of fruits and vegetables OR: 1.22, 95% CI: 1.04–1.42). In addition, supplementary analysis showed that the higher the level of physical activity the more increased the ORs of healthy ageing compared to normal ageing or death (very physically active OR: 9.64, 95% CI: 5.62–16.55; fairly OR: 8.20, 95% CI: 4.77–14.12; not very OR: 4.20, 95% CI: 2.42–7.31; not at all: reference category). The same trend was also observed for the vegetable and fruits consumption (more than six servings OR: 1.51, 95% CI: 1.23–1.85; three to six servings OR: 1.16, 95% CI: 0.99–1.36; less than two servings: reference category). Smoking and alcohol consumption had no significant associations.

To examine whether our findings differed between men and women, we also performed our analyses separately for these two subpopulations. The observed relationships between lifestyle behaviours and healthy ageing compared to normal ageing or death did not change (Supplementary File S4). In addition, to examine whether our conclusions were influenced by the categorisation of participants as healthy agers based on the two highest quintiles of the healthy ageing score, we also performed our analyses by using a different categorisation. We performed our analyses by considering participants scoring in the three highest quintiles of the healthy ageing score distribution as healthy agers (i.e., 59.70–100 scores) and compared them against normal agers (i.e., 0–59.69 scores) or dead participants. Our conclusions regarding the protective effect of physical activity and daily consumption of fruits and vegetables did not alter (Supplementary File S5).

## 4. Discussion

In this study, we investigated the associations of four healthy lifestyle behaviours with healthy ageing and survival from a large dataset (n = 10,900) of five LAC countries (Dominican Republic, Cuba, Peru, Puerto Rico and Mexico). Participants engaging in physical activity and in a diet with daily consumption of fruits and vegetables were individually associated with increased odds of healthy ageing and survival. In addition, we found that the more physically active the participants and the higher the number of fruits and vegetables servings, the higher the odds of ageing healthily. Never smoking and moderate alcohol consumption were not individually associated with healthy ageing but all these four behaviours in combination had a positive effect both for healthy ageing and survival.

The world’s older population is increasing at a higher pace than the total population, and the region of LAC exhibits the highest growth rate of the 60-and-older population from 2015 to 2020 [29]. Although research on healthy ageing has been growing, there is still limited research available for LAC [2]. Our study is among the first reporting a beneficial association of lifestyle habits with healthy ageing or survival in a LAC dataset. These findings are in accordance with those from other studies, which examined lifestyle behaviours and adverse health outcomes in older age, in datasets from high-and-middle income countries [9,14,30]. Physical activity seems to be related with an increased healthy life expectancy by preventing many chronic diseases (i.e., arterial hypertension, diabetes mellitus type 2, store, cancer) [31], and/or age-related diseases (i.e., dementia and Alzheimer’s disease) [32]. The complex biophysiological pathway between physical activity and healthy ageing and survival is yet to be solved. However, this could be partly explained by the favourable biomarker profile of physically active people; higher cell endurance, muscle tissue functionality and energy metabolism [33], reduced fat mass and adipose tissue inflammation [34]. Other studies have also reported a beneficial association of fruits and vegetables consumption with healthy ageing [35,36]. Research has shown that adherence to Mediterranean diet, which includes high consumption of fruits and vegetables, is associated with decreased risk of all-cause mortality, better health outcomes in older people and a reduced risk of frailty [37]. The high antioxidant capacity of fruits and vegetables along with their high fibre and vitamins content could possibly explain this beneficial association [38]. In addition, fruits and vegetables are sources of phytochemicals—the bioactive non-nutrient plant compound—which could contribute to the prevention or minimization of oxidative stress caused by free radicals and thus decrease the risk of chronic diseases [39]. For instance, carotenoids, polyphenols, saponins and phytoestrogens are among those phytochemicals which have shown anticarcinogenic, antioxidative and immunomodulatory effects among others [40].

In regards to moderate alcohol consumption and never smoking, our study did not indicate an individual significant association with healthy ageing. A recent meta-analysis has shown that moderate alcohol consumption seems to be positively associated with healthy ageing but in general associations are mixed [15]. The latest report on the alcohol burden of disease also did not replicate the finding that moderate consumption of alcohol is beneficial; a zero level of alcohol consumption constitutes the level that minimises health loss in the total population [41]. Nevertheless, other studies have found a protective effect of light-to-moderate alcohol consumption against functional health decline among middle-aged people [42]. However these findings should be interpreted with caution, as the beneficial effects of the low to moderate drinking groups (compared with non-drinkers) could be biased by the poor health of former drinkers that are also included in the non-drinkers group in many studies [43,44]. The non-significant result of smoking could be attributed to the limited period of follow-up time (i.e., 4 years) or the relatively older cohort. Studies, which include an older sample, are biased towards those smoking and surviving compared to those that have never smoked and died (survival bias) [45]. Previous meta-analysis also confirms that the negative effects of smoking are more pronounced in younger cohorts [15]. When considering all behaviours in combination, we found that the higher the number of healthy lifestyle behaviours that a participant engaged in during the baseline, the more increased the likelihood of healthy ageing and survival in the follow-up. This comes in agreement with previous research within a high-income setting [9].

A prolonged life longevity is accompanied by multimorbidity [46] which as a consequence poses an extra burden in health systems. As LAC area already exhibits high life expectancy and is the one with the fastest estimated ageing growth, major concerns are raised about the need for early non-pharmacological measures and interventions in older adults to promote healthy ageing. Our findings reflect the importance of healthy lifestyle behaviours and point out the public health actions that must be preserved and prioritised. Adoption of healthy behavioural habits could contribute to the improvement of well-being of the older populations in LACs. Nevertheless, other socioeconomic factors should also be considered when healthy ageing interventions are suggested. For instance, a study with data from 32 countries indicated that overall economic development of the region, public health expenditure and sanitation facilities considerably relate to healthy ageing [47].

### Strengths and Limitations

Based on our knowledge, this is the first study, including a large sample, which examined healthy ageing within the functional ability framework and lifestyle behaviour in LAC countries. Our results contribute to the advance of healthy ageing knowledge in under-examined areas and especially in LMICs as current research has mostly focused on other countries [2]. The limited attrition between baseline assessment and follow-up together with our further analyses, which indicated that in most of the cases participants included in the study did not differ with those excluded, limit any potential information bias. In addition, the observed heterogeneity (I^2^) was minimal in most cases, reflecting the limited variability in point estimates among countries.

In our study, we created a healthy ageing index within the functional ability framework, as it is the latest recommendation from the WHO [8]. Previous research has mostly focused on the presence or not of some disease/illness as a recent review indicated [7]. Yet, the functioning framework is considered more useful for effective public-health responses than considering specific diseases [8]. For this reason, we created this healthy ageing index by not taking into account specific conditions (i.e., depression, cardiovascular diseases, etc.). Furthermore, we did not consider other domains of healthy ageing (i.e., psychological well-being and social well-being) [48] since the healthy ageing model provided by WHO also incorporates “resilience” as an ability to maintain or improve the level of functioning during challenging periods. This suggests that older people could use psychological/physiological resources and environmental characteristics (i.e., social relationships) to maintain or improve their functional ability. However, these domains were not included in our index, as our ultimate intention was to build an index focusing on the final observed outcome -functional ability-.

Our findings should be interpreted within the context of this study limitations. Lifestyle behaviours were all assessed via self-reported questionnaires. Therefore, potential measurement error could have occurred. Furthermore, questions were too broad not allowing to holistically assess the impact of different frequencies and intensities in physical activity, or of specific fruits and vegetables that were consumed. In addition, even though interviewers underwent substantial training to ensure consistency in the way surveys were conducted in the various settings, we cannot exclude the bias of cross-cultural differences among countries in the conceptualisation of some questions. Another limitation of our study could be the quite arbitrary way of categorising participants who are in the two highest fifths of the baseline healthy ageing score distribution as healthy agers and all others as normal agers. Nevertheless, supplementary analyses (Supplementary File S5) indicated that our conclusions were not influenced by a different categorisation of participants in the health and normal ageing groups. In addition, we characterised healthy behaviour when participants were “very” or “fairly” physically active and when they consumed fruits and vegetables on a daily basis. These categorisations constitute assumptions of our study and consequently our findings should be interpreted within the limitations caused by these. The limited follow-up time inherited with these data also made it impossible to examine the impact of lifestyle behaviours in a more prolonged time. Finally, our sample included participants 65 years old and over. Hence, we were unable to assess lifestyle behaviour and examine healthy ageing within a life course perspective approach [49].

## 5. Conclusions

Physical activity and a diet rich in fruits and vegetables seem to be associated with healthy ageing and survival. Even though never smoking and moderate alcohol consumption were not significantly associated, once we combine all four healthy behaviours their effect is substantial. Furthermore, the more the healthy lifestyle behaviours adopted, the higher the odds of being old and maintaining well-being. Our results could help in the establishment of policies promoting a healthy lifestyle in LAC countries. Future studies evaluating physical activity, nutritional and other lifestyle interventions within the LAC area are needed to attain a better ageing process for these populations.

## Figures and Tables

**Table 1:** Baseline characteristics of participants in the healthy and normal ageing group * in the follow-up wave.

Country	Cuba		Dominican Republic		Mexico		Peru		Puerto Rico	
Characteristic	Healthy Ageing (n = 494)	Normal Ageing (n = 1513)	Healthy Ageing (n = 234)	Normal Ageing (n = 963)	Healthy Ageing (n = 153)	Normal Ageing (n = 1309)	Healthy Ageing (n = 579)	Normal Ageing (n = 732)	Healthy Ageing (n = 188)	Normal Ageing (n = 1077)
Age (mean, SD)	70.9 ± 4.61	74.87 ± 6.65 †	70.89 ± 5.17	74.7 ± 6.97 †	69.9 ± 4.00	74.2 ± 6.35 †	71.1 ± 5.20	76.7 ± 7.21 †	72.0 ± 4.56	75.7 ± 6.65 †
Gender-Female	291 (58.9%)	1041 (68.8%) †	140 (60.1%)	689 (71.6%) †	99 (64.7%)	845 (64.6%)	343 (59.2%)	474 (64.8%) †	141 (75.0%)	728 (67.8%)
Education Level-above primary	440 (89.4%)	1099 (72.7%) †	101 (43.2%)	259 (27.1%) †	87 (57.2%)	355 (27.1%) †	493 (86.2%)	559 (77.0%) †	164 (87.2%)	846 (78.9%) †
Never smoking	244 (49.6%)	589 (56.9%) †	124 (53.0%)	523 (54.4%)	91 (59.5%)	913 (69.7%) †	471 (81.8%)	625 (85.9%) †	150 (79.8%)	789 (73.5%)
Moderate alcohol consumption	53 (10.9%)	140 (9.4%)	19 (8.3%)	52 (5.4%)	30 (19.7%)	225 (17.3%)	21 (3.7%)	30 (4.2%)	21 (11.2%)	92 (8.6%)
Physical activity (very or fairly)	425 (86.6%)	1047 (69.4%) †	188 (80.7%)	626 (65.3%) †	133 (86.9%)	841 (64.6%) †	471 (81.6%)	462 (63.6%) †	176 (93.6%)	722 (67.3%) †
Fruits/vegetable consumption (≥3 servings in the last 3 days)	409 (83.1%)	1130 (74.8%) †	138 (59.0%)	460 (48.7%) †	116 (76.3%)	931 (71.5%)	477 (83.1%)	593 (82.4%)	156 (83.0%)	807 (75.3%) †

Notes: SD: standard deviation; †: *p*-value of the *t* test or the χ^2^ < 0.05. * healthy ageing group includes those participants who in the follow-up assessment were in the two highest fifths of the healthy ageing score distribution (i.e., better health level) and normal ageing group those who were in the three lowest fifths; percentages in the parentheses are calculated in the non-missing cases in the covariates.

**Table 2:** Associations between lifestyle behaviours, healthy ageing and survival.

	Healthy Ageing * Adjusted OR ^†^ (95% CI)			
Country/Lifestyle Behaviour	Physical Activity (Very or Fairly vs. Not Very or Never)	Smoking (Never vs. Former or Current)	Drinking Behaviour (Moderate Drinking vs. Never or Heavy Drinking)	Fruits/Vegetables Consumption (≥3 Servings in the Last Three Days vs. <3)
Cuba	2.56 (1.93–3.41)	0.97 (0.77–1.24)	0.83 (0.57–1.20)	1.41 (1.06–1.87)
Dominican Republic	2.11 (1.46–3.04)	1.03 (0.75–1.40)	1.21 (0.68–2.17)	1.35 (0.99–1.83)
Mexico	2.98 (1.81–4.93)	0.58 (0.37–0.90)	1.09 (0.66–1.81)	1.11 (0.73–1.68)
Peru	2.32 (1.75–3.08)	0.90 (0.62–1.30)	0.70 (0.34–1.44)	0.92 (0.66–1.29)
Puerto Rico	6.30 (3.43–11.56)	1.30 (0.85–1.97)	1.80 (1.02–3.17)	1.44 (0.94–2.21)
Pooled Effect	2.59 (2.20–3.03)	0.95 (0.82–1.10)	1.04 (0.82–1.30)	1.24 (1.06–1.44)
I^2^; *p*-value	61.3%; 0.035	44.7%; 0.124	38.0%; 0.168	16.6%; 0.309
	Survival * **Adjusted OR ^†^ (95% CI)**			
Cuba	2.29 (1.86–2.82)	1.16 (0.92–1.45)	1.04 (0.74–1.46)	1.15 (0.92–1.44)
Dominican Republic	1.82 (1.43–2.30)	1.18 (0.93–1.50)	1.36 (0.78–2.37)	0.94 (0.75–1.19)
Mexico	2.08 (1.50–2.86)	0.94 (0.61–1.44)	1.16 (0.74–1.80)	1.17 (0.84–1.64)
Peru	2.75 (1.90–3.63)	1.20 (0.70–2.04)	0.72 (0.33–1.60)	1.06 (0.66–1.73)
Puerto Rico	2.71 (2.02–3.63)	1.34 (0.95–1.89)	1.24 (0.73–2.09)	1.45 (1.08–1.96)
Pooled Effect	2.23 (1.98–2.52)	1.17 (1.02–1.34)	1.11 (0.90–1.37)	1.13 (0.99–1.28)
I^2^; *p*-value	34.4%; 0.192	0.0%; 0.807	0.0%; 0.737	21.9%; 0.275

Notes: CI: confidence interval; OR: Odds ratio; I^2^: heterogeneity. *: healthy ageing compared to normal ageing or death; survival (healthy or normal ageing) compared to death; number of participants per country: Cuba: 2557, Dominican Republic: 1608, Mexico: 1633, Peru: 1392, Puerto Rico: 1554. ^†^: models are adjusted for age, gender, education level and all other lifestyle behaviour variables.

**Table 3:** Associations between number of healthy behaviours, healthy ageing and survival.

Country	Healthy Ageing *			
	Adjusted OR ^†^ (95% CI)			
	Number of Healthy Behaviours Compared to Zero or One			
	Two	Three	Four	Two-Four
Cuba	1.67 (1.23–2.29)	2.12 (1.54–2.92)	1.74 (0.77–3.96)	1.86 (1.39–2.49)
Dominican Republic	1.64 (1.16–2.32)	1.98 (1.32–2.96)	2.48 (0.78–7.86)	1.77 (1.28–2.43)
Mexico	1.29 (0.74–2.24)	1.31 (0.74–2.33)	2.75 (1.04–7.26)	1.35 (0.80–2.29)
Peru	0.98 (0.64–1.51)	1.64 (1.08–2.49)	1.14 (0.21–6.22)	1.31 (0.88–1.96)
Puerto Rico	1.63 (0.84–3.17)	4.21 (2.25–7.87)	4.93 (1.77–13.71)	2.92 (1.60–5.36)
Pooled Effect	1.46 (1.22–1.76)	2.00 (1.65–2.42)	2.46 (1.54–3.92)	1.72 (1.45–2.05)
I^2^; *p*-value	16.0%; 0.313	53.0%; 0.074	0.0%; 0.509	31.5%; 0.211
	Survival *			
	**Adjusted OR ^†^ (95% CI)**			
Cuba	1.57 (1.24–2.00)	2.17 (1.66–2.85)	3.53 (1.21–10.28)	1.80 (1.44–2.24)
Dominican Republic	1.45 (1.13–1.88)	1.71 (1.22–2.40)	1.80 (0.48–6.72)	1.53 (1.21–1.93)
Mexico	1.79 (1.24–2.60)	2.19 (1.42–3.36)	2.13 (0.79–5.68)	1.94 (1.37–2.75)
Peru	1.52 (0.90–2.55)	2.91 (1.66–5.12)	1.80 (0.26–12.22)	2.02 (1.22–3.34)
Puerto Rico	1.84 (1.33–2.54)	3.44 (2.34–5.05)	4.11 (1.18–14.28)	2.35 (1.73–3.18)
Pooled Effect	1.60 (1.40–1.84)	2.29 (1.94–2.69)	2.64 (1.53–4.54)	1.83 (1.61–2.08)
I^2^; *p*-value	0.0%; 0.790	50.3%; 0.090	0.0%; 0.840	22.0%; 0.274

Notes: CI: confidence interval; OR: Odds ratio; I^2^: heterogeneity. *: healthy ageing compared to normal ageing or death; survival (healthy or normal ageing) compared to death; number of participants per country: Cuba: 2557, Dominican Republic: 1608, Mexico: 1633, Peru: 1392, Puerto Rico: 1554. ^†^: models are adjusted for age, gender, education level and all other lifestyle behaviour variables.

## Supplementary Materials

The following are available online at http://www.mdpi.com/2072-6643/10/11/1593/s1. Supplementary File S1: Healthy ageing questions origin; Supplementary File S2: Development of a healthy ageing index; Supplementary File S3: Participants’ (included and excluded from the analyses) baseline characteristics; Supplementary File S4: Associations between lifestyle behaviours and healthy ageing per gender; Supplementary File S5: Associations between lifestyle behaviours and healthy ageing-different categorisation of healthy ageing.
