# Supplementary material for: Associations of Lifestyle Behaviour and Healthy Ageing in Five Latin American and the Caribbean Countries—A 10/66 Population-Based Cohort Study
Source: Nutrients. 2018 Oct 30;10(11):1593. doi: 10.3390/nu10111593 (PMC6266391; doi:10.3390/nu10111593)
Supplement: Supplementary file 1 [file nutrients-10-01593-s001.zip › Supplementary File S1.pdf]

Table A1.1 Healthy Ageing Questions Origin.

Items/Questions	Origin	Questionnaire
household responsibilities difficulty	participant	WHO-DAS II
walking a km difficulty	participant	WHO-DAS II
washing whole body difficulty	participant	WHO-DAS II
getting dressed difficulty	participant	WHO-DAS II
carrying out work & everyday activities difficulty	participant	WHO-DAS II
making decisions difficulty	informant	CSI'D'-RELSCORE
using the toilet difficulty	informant	CSI'D'-RELSCORE
handling money difficulty	informant	CSI'D'-RELSCORE
hearing problem	participant & informant	Health (including pain and impairments)
eye problem	participant & informant	Health (including pain and impairments)
finding right word difficulty	informant	CSI'D'-RELSCORE
change in daily activities	informant	CSI'D'-RELSCORE
forgets where he/she is	informant	CSI'D'-RELSCORE
difficulty completing chores	informant	CSI'D'-RELSCORE
sleep trouble or recent change in pattern	participant	Mental Health (GMS-version B3)
feeling of not coping properly with everyday routine	participant	Mental Health (GMS-version B3)
gets worn out or exhausted during daytime or evening	participant	Mental Health (GMS-version B3)
time in seconds taken to walk 10 metres	clinical examination	neurological assessment (NEUROEX)
learn test	participant	10 words list learning

delayed recall	participant	CSI'D'
long memory test	participant	CSI'D'
immediate recall	participant	CSI'D'
verbal fluency	participant	CSI'D'
time orientation	participant	CSI'D'
praxis-fold a piece of paper	participant	CSI'D'
story recall difficulty	participant	CSI'D'

WHO-DAS II: World Health Organization. Disabilty Assessment Schedule 2.0; CSI'D'-RELSCORE: Community Screening Interview for Dementia-Informant Scale; GMS: Geriatric Mental State Interview; NEUROEX: Neurological Examination; CSI'D': Community Screening Interview for Dementia.
