# Supplementary material for: Associations of Lifestyle Behaviour and Healthy Ageing in Five Latin American and the Caribbean Countries—A 10/66 Population-Based Cohort Study
Source: Nutrients. 2018 Oct 30;10(11):1593. doi: 10.3390/nu10111593 (PMC6266391; doi:10.3390/nu10111593)
Supplement: Supplementary file 1 [file nutrients-10-01593-s001.zip › Supplementary File S2.pdf]

Development of a healthy ageing index

We performed the data-driven methodology of Exploratory Factor Analysis (EFA) to identify the appropriate number of factors and pattern of relationships among the 26 items (**Supplementary File 1**).[1] Most items were categorical and for those that were given in a continuous scale (i.e. instant recall, verbal fluency, speed test, story recall), we divided the whole sample in three groups according to the lower and upper quartiles of each distribution; values below the 25th percentile, between the 25th and 75th and above the 75th indicated high, moderate and low performance, respectively.

To perform the EFA we used a 30% random sample of our sample. We used mean and variance-adjusted weighted least-squares (WLSMV) estimator, which is suitable for the analysis of categorical data, polychoric correlations and parsimax (oblique) rotation allowing for correlations among factors.[2] After examination of eigenvalues (12.53, 2.40, 1.74, 1.18), goodness-of-fit statistics and interpretability of factor structure, the four-factor solution was the best solution (χ^2=679.78, df=227, RMSEA=0.028; 90%CI=0.026-0.030, CFI=0.994).[3]

Table A2.1 provides the results of the EFA analysis and in Table A2.2 factor correlations are illustrated.

Table A2.1 Exploratory factor analysis standardised loadings-Parsimax Rotation.

Questions/Items	Factor 1	Factor 2	Factor 3	Factor 4
household responsibilities difficulty	**0.66**	0.29	0.33	-0.15
walking a km difficulty	**0.69**	0.07	0.31	-0.06
washing whole body difficulty	**0.90**	-0.01	0.02	0.21
getting dressed difficulty	**0.87**	0.05	0.02	0.17
carrying out work & everyday activities difficulty	**0.64**	0.35	0.29	-0.14
making decisions difficulty	0.14	**0.74**	-0.03	0.25
using the toilet difficulty	**0.52**	0.32	0.03	0.31
handling money difficulty	0.18	**0.63**	0.02	0.31
hearing problem	0.09	-0.13	**0.43**	0.21
eye problem	0.09	-0.20	**0.47**	0.10
finding right word difficulty	-0.03	**0.66**	0.19	0.14
change in daily activities	0.06	**0.72**	0.23	0.02
forgets where he/she is	0.20	**0.58**	0.03	0.35
difficulty completing chores	0.07	**0.67**	0.07	0.25
sleep trouble or recent change in pattern	-0.10	-0.08	**0.67**	-0.11
feeling of not coping properly with everyday routine	0.04	0.17	**0.54**	0.06
gets worn out or exhausted during daytime or evening	-0.17	-0.13	**0.82**	0.01
time in seconds taken to walk 10 metres	**0.33**	-0.18	0.13	0.12
learn test	0.06	0.19	0.08	**0.55**
delayed recall	0.06	0.14	0.09	**0.38**
long memory test	0.04	0.05	0.13	**0.76**
immediate recall	0.15	0.20	0.05	**0.43**
verbal fluency	0.09	0.11	0.14	**0.60**
time orientation	0.08	0.22	0.14	**0.62**
praxis-fold a piece of paper	0.21	0.15	0.02	**0.41**
story recall difficulty	-0.06	0.15	0.14	**0.60**

Notes. χ^2. 679.78, df: 227, p-value<0.05; Comparative Fit Index (CFI): 0.994; Root Mean Square Error of Approximation (RMSEA): 0.028, 90%CI:0.026-0.030.

.**Table A2.2** Factor correlations from Exploratory Factor Analyses.

	Factor 1	Factor 2	Factor 3	Factor 4
Factor 1	1			
Factor 2	0.485*	1		
Factor 3	0.335*	0.269*	1	
Factor 4	0.344*	0.551*	0.225*	1

*: significant in α=0.05.

We performed Confirmatory Factor Analysis (CFA) and we built a bifactor model as our ultimate goal was to create a measurement model reflecting the latent (unobserved) construct of healthy ageing.[3] A bifactor model allows having one general factor (i.e. the healthy ageing index) in which all items load and sub-domain (domain-specific or group) factors; the latter represent variability not explained by the general factor.[5] As a consequence, a bifactor model can assist in judging the practical unidimensionality of a scale where a dominant factor exists together with other smaller domain-specific factors due to similar content.[6]

As EFA suggested, we used four sub-domain factors. Once the bifactor model was identified (Table A3), we also calculated psychometric coefficients of the bifactor structure: omega hierarchical (ω_H) coefficients and omega hierarchical subscales (ω_{HS}).[7] A high ω_H value (>0.80) in the bifactor structure indicates that the general factor is the dominant source of systematic variance with sub-domain factors having less influence; ω_{HS} represents the proportion of reliable systematic variance of a subscale score after partitioning out general factor variability. We found: ω_H = 0.85; ω_{HS1} = 0.05; ω_{HS2} = 0.01; ω_{HS3} = 0.02; ω_{HS4} = 0.02. Based on our findings, 85% of the variance of the total scale was attributable to the general factor only. Hence, raw scores can be considered as indicators of the healthy ageing factor and are marginally affected by the sub-domain factors. Even though some items exhibited stronger loadings onto domain-specific factors compared to the general factor, the reliability of the former was limited once we accounted for the general healthy ageing factor (i.e. ω_{HS1} = 0.05; ω_{HS2} = 0.01; ω_{HS3} = 0.02; ω_{HS4} = 0.02). As coefficient omega hierarchical subscales indicated that subscales have limited added value once we accounted for the general factor, we proceeded in our analyses by only considering the general factor.

Table A2.3 Standardised loadings-Bifactor model results.

Items/ Questions	General Factor	Sub-domain Factor 1	Sub-domain Factor 2	Sub-domain Factor 3	Sub-domain Factor 4
household responsibilities difficulty	0.66	0.66			
walking a km difficulty	0.57	0.62			
washing whole body difficulty	0.75	0.65			
getting dressed difficulty	0.75	0.62			
carrying out work & everyday activities difficulty	0.69	0.64			
making decisions difficulty	0.90		0.24		
using the toilet difficulty	0.88	0.25			
handling money difficulty	0.95		0.15		
hearing problem	0.31				0.35
eye problem	0.24				0.41
finding right word difficulty	0.75		0.25		
change in daily activities	0.75		0.37		
forgets where he/she is	0.96		-0.03		
difficulty completing chores	0.86		0.30		
sleep trouble or recent change in pattern	0.13				0.63
feeling of not coping properly with everyday routine	0.51				0.41
gets worn out or exhausted during daytime or evening	0.18				0.65
time in seconds taken to walk 10 metres	0.24	0.26			
learn test	0.65			0.33	
delayed recall	0.47			0.21	
long memory test	0.67			0.49	
immediate recall	0.64			0.23	
verbal fluency	0.70			0.31	
time orientation	0.76			0.40	
praxis-fold a piece of paper	0.59			0.23	
story recall difficulty	0.56			0.45	

Notes. χ^2: 4039.14, df: 273, p-value<0.05; Comparative Fit Index (CFI): 0.985; Root Mean Square Error of Approximation (RMSEA): 0.040, 90%CI:0.039-0.041.

References

1. Brown, T.A.; Confirmatory Factor Analysis for Applied Research, 2nd ed.; The Guilford Press; New York, London, 2015.
2. Muthén, L. K.; Muthén, B. O., Mplus User's Guide. 7th ed.; Muthén & Muthén: Los Angeles, CA, 1998-2015.
3. Yang, Y.; Xia, Y., On the number of factors to retain in exploratory factor analysis for ordered categorical data. Behavior research methods 2015, 47 (3), 756-72. 10.3758/s13428-014-0499-2.
4. Reise, S. P.; Bonifay, W. E.; Haviland, M. G., Scoring and modeling psychological measures in the presence of multidimensionality. J Pers Assess 2013, 95 (2), 129-40. 10.1080/00223891.2012.725437.
5. Reise, S. P.; Moore, T. M.; Haviland, M. G., Bifactor models and rotations: exploring the extent to which multidimensional data yield univocal scale scores. J Pers Assess 2010, 92 (6), 544-59. 10.1080/00223891.2010.496477.
6. Chen, F.F.; West, S.G.; Sousa, K.H., A Comparison of Bifactor and Second-Order Models of Quality of Life. Multivariate Behav Res, 2006. 41(2), 189-225. 10.1207/s15327906mbr4102_5.
7. Rodriguez, A.; Reise, S. P.; Haviland, M. G., Evaluating bifactor models: Calculating and interpreting statistical indices. Psychol Methods 2016, 21 (2), 137-150. 10.1037/met0000045.
