# Supplementary material for: Associations of Lifestyle Behaviour and Healthy Ageing in Five Latin American and the Caribbean Countries—A 10/66 Population-Based Cohort Study
Source: Nutrients. 2018 Oct 30;10(11):1593. doi: 10.3390/nu10111593 (PMC6266391; doi:10.3390/nu10111593)
Supplement: Supplementary file 1 [file nutrients-10-01593-s001.zip › Supplementary File S3.pdf]

Table A3.1: Baseline characteristics of participants included in our analyses and those excluded due to lost to follow-up survey or refused to participate.

**Country**	**Cuba**			**Dominican Republic**			**Mexico**		
Characteristics	study participants (n= 2,615)	not traced/ refused (n=329)	missing data*	study participants (n= 1,664)	not traced/ refused (n=347)	missing data	study participants (n= 1,671)	not traced/ refused (n=332)	missing data
Age (mean, SD)	75.2 ± 7.08	74.1 ± 6.63 †	0.3%	75.4 ± 7.57	74.6 ± 7.19	0.0%	74.3 ± 6.61	74.5 ± 6.91	0.0%
Gender-Female	1697 (64.9%)	216 (65.7%)	0.0%	1104 (66.4%)	221 (63.7%)	0.1%	1063 (63.6%)	205 (61.7%)	0.0%
Education Level-Completed primary	873 (33.5%)	106 (32.2%)	0.3%	295 (17.9%)	75 (21.7%)	1.1%	294 (17.6%)	57 (17.2%)	0.1%
Ever smoked-no	1421 (54.5%)	194 (59.1%)	0.3%	875 (52.6%)	174 (50.1%)	0.1%	1142 (68.3%)	235 (70.8%)	0.0%
Drinking behaviour (moderate)	247 (9.6%)	24 (7.5%)	1.6%	95 (5.8%)	24 (6.9%)	1.0%	290 (17.5%)	62 (18.8%)	0.8%
Physical activity (very and fairly)	1757 (67.5%)	220 (67.3%)	0.5%	1040 (62.8%)	224 (64.6%)	0.4%	1063 (64.0%)	228 (69.5%)	0.7%
Fruits/vegetables consumption	1965 (75.4%)	218 (66.3%) †	0.3%	834 (50.9%)	191 (55.7%)	1.5%	1184 (71.4%)	232 (70.9%)	0.7%

**Country**	**Peru**			**Puerto Rico**		
Characteristics	study participants (n= 1,463)	not traced/ refused (n=470)	missing data	study participants (n= 1,563)	not traced/ refused (n=446)	missing data
Age (mean, SD)	74.9 ± 7.48	74.4 ± 6.98	0.0%	76.3 ± 7.23	76.6 ± 8.07	0.0%
Gender-Female	886 (60.6%)	297 (63.2%)	0.0%	1047 (67.2%)	300 (67.6%)	0.3%
Education Level-Completed primary	556 (38.4%)	171 (36.5%) †	1.0%	305 (19.5%)	110 (24.7%)	0.4%
Ever smoked-no	1216 (83.6%)	385 (81.9%)	0.5%	1131 (72.6%)	323 (72.7%)	0.3%
Drinking behaviour (moderate)	63 (4.4%)	21 (4.6%)	2.7%	141 (9.1%)	46 (10.4%)	0.5%
Physical activity (very and fairly)	996 (68.6%)	338 (72.1%)	0.8%	1009 (64.8%)	269 (60.9%)	0.3%
Fruits/vegetables consumption	1189 (82.5%)	356 (76.2%) †	1.4%	1155 (74.2%)	312 (70.3%)	0.4%

Notes. SD: standard deviation; †: p value of the t test or the $\chi^2 < 0.05$; *: missing data on the characteristics in the sample of study participant.
