# Supplementary material for: Associations of Lifestyle Behaviour and Healthy Ageing in Five Latin American and the Caribbean Countries—A 10/66 Population-Based Cohort Study
Source: Nutrients. 2018 Oct 30;10(11):1593. doi: 10.3390/nu10111593 (PMC6266391; doi:10.3390/nu10111593)
Supplement: Supplementary file 1 [file nutrients-10-01593-s001.zip › Supplementary File S4.pdf]

Table A4.1. Associations between lifestyle behaviours and healthy ageing per gender.

**Men**	**Healthy Ageing***	
Lifestyle Behaviour	Adjusted OR † (95% CI)	I^2; p-value
**Physical Activity**		
not very or never	Reference category	
very or fairly	2.30 (1.78-2.98)	59.5%; 0.042
**Smoking**		
former or current	Reference category	
never	0.84 (0.67-1.06)	0.0%; 0.806
**Drinking behaviour**		
no or heavy drinking	Reference category	
moderate drinking	1.01 (0.77-1.32)	27.3%; 0.240
**Consumption of fruits and vegetables**		
< 3 in the last 3 days	Reference category	
more than 3 in the last 3 days	1.23 (0.96-1.57)	0.0%; 0.751

**Women**	**Healthy Ageing***	
Lifestyle Behaviour	Adjusted OR † (95% CI)	I^2; p-value
**Physical Activity**		
not very or never	Reference category	
very or fairly	2.70 (2.21-3.31)	53.2%; 0.073
**Smoking**		
former or current	Reference category	
never	1.04 (0.85-1.27)	31.7%; 0.210
**Drinking behaviour**		
no or heavy drinking	Reference category	
moderate drinking	1.15 (0.76-1.74)	52.8%; 0.076
**Consumption of fruits and vegetables**		
< 3 in the last 3 days	Reference category	
more than 3 in the last 3 days	1.25 (1.03-1.51)	0.0%; 0.448

Notes: CI: confidence interval; OR: Odds ratio; *: healthy ageing compared to normal ageing or death; †: models are adjusted for age, gender, education level and all other lifestyle behaviour variables.
