# Supplementary material for: Associations of Lifestyle Behaviour and Healthy Ageing in Five Latin American and the Caribbean Countries—A 10/66 Population-Based Cohort Study
Source: Nutrients. 2018 Oct 30;10(11):1593. doi: 10.3390/nu10111593 (PMC6266391; doi:10.3390/nu10111593)
Supplement: Supplementary file 1 [file nutrients-10-01593-s001.zip › Supplementary File S5.pdf]

Table A5.1. Associations between lifestyle behaviours and healthy ageing.

**Country/ Lifestyle Behaviour**	**Healthy Ageing*** **Adjusted OR† (95% CI)** **Physical Activity** (very or fairly compared to not very or never)	**Smoking** (never compared to former or current)	**Drinking behaviour** (moderate drinking compared to never or heavy drinking)	**Consumption of fruits-vegetables** (three or more than three servings in the last three days compared to less than three)
Cuba	2.84 (2.28-3.52)	1.02 (0.83-1.25)	0.98 (0.72-1.34)	1.49 (1.19-1.87)
Dominican Republic	2.16 (1.64-2.85)	1.34 (1.04-1.72)	1.53 (0.94-2.47)	1.31 (1.03-1.68)
Mexico	2.47 (1.79-3.40)	0.72 (0.52-1.00)	1.05 (0.73-1.50)	1.25 (0.94-1.68)
Peru	3.14 (2.40-4.12)	1.30 (0.89-1.89)	0.84 (0.41-1.72)	0.92 (0.65-1.30)
Puerto Rico	3.56 (2.62-4.82)	1.29 (0.95-1.76)	1.28 (0.83-1.97)	1.70 (1.27-2.29)
Pooled Effect	2.79 (2.48-3.15)	1.10 (0.98-1.25)	1.11 (0.92-1.33)	1.35 (1.20-1.52)
I^2; p-value	43.4%; 0.132	64.0%; 0.025	0.0%; 0.493	50.7%; 0.088

Notes: CI: confidence interval; OR: Odds ratio. *: healthy ageing compared to normal ageing or death; healthy ageing group includes those participants who in the follow-up assessment were in the three highest fifths of the healthy ageing score distribution (i.e. better health level) and normal ageing group those who were in the two lowest fifths †: models are adjusted for age, gender, education level and all other lifestyle behaviour variables.
